# Respiratory Monitoring Based on Tracheal Sounds: Continuous Time-Frequency Processing of the Phonospirogram Combined with Phonocardiogram-Derived Respiration

**DOI:** 10.3390/s21010099

**Published:** 2020-12-25

**Authors:** Xinyue Lu, Christine Azevedo Coste, Marie-Cécile Nierat, Serge Renaux, Thomas Similowski, David Guiraud

**Affiliations:** 1 Faculté des Sciences, University of Montpellier, F-34090 Montpellier, France; sindayue@gmail.com; 2 NeuroResp, F-34600 Les Aires, France; serge.renaux@neuroresp.com; 3 INRIA, F-34090 Montpellier, France; david.guiraud@inria.fr; 4 UMRS1158 Neurophysiologie Respiratoire Expérimentale et Clinique, INSERM, Sorbonne Université, F-75005 Paris, France; m-cecile.nierat@wanadoo.fr (M.-C.N.); thomas.similowski@upmc.fr (T.S.); 5 NEURINNOV, F-34090 Montpellier, France; 6 AP-HP, Site Pitié-Salpêtrière, Service de Pneumologie, Médecine Intensive et Réanimation (Département R3S), Groupe Hospitalier Universitaire APHP-Sorbonne Université, F-75013 Paris, France

**Keywords:** respiratory monitoring, breathing detection, tracheal sounds, Phonocardiogram-Derived Respiration (PDR)

## Abstract

Patients with central respiratory paralysis can benefit from diaphragm pacing to restore respiratory function. However, it would be important to develop a continuous respiratory monitoring method to alert on apnea occurrence, in order to improve the efficiency and safety of the pacing system. In this study, we present a preliminary validation of an acoustic apnea detection method on healthy subjects data. Thirteen healthy participants performed one session of two 2-min recordings, including a voluntary respiratory pause. The recordings were post-processed by combining temporal and frequency detection domains, and a new method was proposed—Phonocardiogram-Derived Respiration (PDR). The detection results were compared to synchronized pneumotachograph, electrocardiogram (ECG), and abdominal strap (plethysmograph) signals. The proposed method reached an apnea detection rate of 92.3%, with 99.36% specificity, 85.27% sensitivity, and 91.49% accuracy. PDR method showed a good correlation of 0.77 with ECG-Derived Respiration (EDR). The comparison of R-R intervals and S-S intervals also indicated a good correlation of 0.89. The performance of this respiratory detection algorithm meets the minimal requirements to make it usable in a real situation. Noises from the participant by speaking or from the environment had little influence on the detection result, as well as body position. The high correlation between PDR and EDR indicates the feasibility of monitoring respiration with PDR.

## 1. Introduction

Impairment of ventilatory control (central respiratory paralysis) may lead to a dependence on external mechanical ventilation (usually with intermittent positive pressure via tracheotomy), even though the main inspiratory effector, the diaphragm, is intact. These may be due to the interruption in the transmission of the control commands (essentially post-traumatic upper cervical spinal cord injury) or abnormalities in the genesis of the control (central, congenital, or acquired alveolar hypoventilation) [1]. An improved treatment, diaphragm pacing, can restore a diaphragmatic contraction and thus relieve patients of the constraint of a mechanical ventilator. Compared to external mechanical ventilation, this technique provides more natural respiration [2,3], improves speeches and olfaction, and helps to reduce infection risks and health care costs [4].

In the context of ventilatory assistance, detecting breathing interruptions resulting from airway obstruction or ventilator disconnection is a fundamental safety issue. All ventilatory assistance devices include built-in flow and/or pressure sensors connected to alarms to alert caregivers of any flow interruption. Implanted phrenic nerve electric stimulators for diaphragm pacing are notable exceptions to this rule. Indeed, they include built-in electronic dysfunction alarms but are devoid of “efficacy” alarms. During diaphragm pacing, an apnea (absence of flow for at least 10 s [5]) due to airway obstruction (obstructive apnea, with respiratory effort) or diaphragmatic neurotransmission failure (central apnea, without respiratory effort) will not result in an immediate alarm if the patient is not equipped with an additional flow sensor [6].

Detecting breathing interruptions is key to diagnosing both central and obstructive apneas. Yet, the usual methods (pneumotachograph, thermistor, etc.) to assess breathing activities and detect apnea episodes rely on flow sensors connected to the airway, which is burdensome and limits their tolerability (imply to wear a mask or a mouthpiece). Notably, monitoring thoracic movements (respiratory effort, with plethysmograph) does not provide an adequate alternative of flow detection because this approach cannot detect obstructive apnoeic events and is sensible to patient’s movements.

The aim of our research was to improve the efficiency and safety of diaphragm pacing systems. We propose to use microphones for monitoring patient’s respiration via tracheal sounds (TS). The approach is non-invasive (no supplementary complexity due to implantation surgery) and miniaturized so that patients can carry the device easily without wearing a mask. It has long been known that ventilatory flow can be accessed transcutaneously by recording TS. Indeed, the turbulent airflow going through the airway causes pressure vibrations that are transmitted to the body surface through the tracheal wall and the surrounding tissues. Tracheal sounds can thus be recorded simply using a skin-taped microphone. They provide a signal that is representative of ventilatory airflow and has been described as “phonospirometry” [7]. A microphone affixed over the suprasternal notch was shown to provide the greatest signal amplitude-to-noise ratio [8]. At this location, cardiac sounds can also be recorded (phonocardiogram).

The present study proposes and seeks to validate firstly signal processing algorithms on healthy subjects. The proposed method is a robust real-time monitoring of respiratory activity through the percutaneous recording of TS. The two main contributions of the methods are: (i) TS embeds temporal and frequency information that, mixed together, enhanced the identification of respiratory phases, and (ii) a new processing of cardiac sound from the TS recording can further provide relevant information about respiratory states.

### 1.1. Apnea Detection

Studies have shown the feasibility of detecting respiration from TS mainly in sleep apnea. The detection algorithms can be divided into two categories:

#### 1.1.1. Detection in the Temporal Domain

Corbishley et al. [8] proposed an algorithm that was based on temporal envelope detection with several user-dependent thresholds and could be embedded in a miniature and low-powered sensing device. Some time later, Yadollahi et al. [9] used the logarithm of TS variance combined with an oximeter to estimate the relative respiratory flow. Recently, Kalkbrenner et al. [10] processed short-term and long-term envelopes of TS combined with measurement of patient movements by IMUs to evaluate the respiratory amplitude changes; however, the processing is offline.

#### 1.1.2. Detection in the Frequency Domain

Chuah et al. [11] proposed the detection of respiratory events using the peak picking technique in a power spectrum limited to specific frequency bands for both chest sounds and TS. Hult et al. [12] improved their own algorithm from Reference [13] using threshold detection in real-time in the frequency envelope of the 300–600 Hz power spectrum. A similar method was used by Kalkbrenner et al. [14], who classified respiratory phases using threshold detection in the 10–600 Hz power spectrum. Kulkas et al. [15] proposed detecting apnea events based on the ratio of the power spectrum density of heart beat (0–50 Hz) to that of heart beat and respiration (0–600 Hz).

Other methods based on artificial neural networks have been proposed, such as by Lei et al. [16]. A complete review of TS processing methods is presented in Muthusamy et al. [17].

As a whole, few methods have been developed for real-time monitoring and even fewer for an ambulatory context. Indeed, most of the studies have been carried out in quiet and controlled acoustic environments with stable sources of noise, and limited participant movements, mainly during sleep. Furthermore, the studies did not incorporate multi-domain detection.

### 1.2. Phonocardiogram (PCG)-Derived Respiration

Electrocardiogram (ECG) corresponds to the electrical potential action of the cardiac muscle. Three principal waves appear together, called the QRS-complex, and are linked to the depolarization of the two ventricles.

During respiration, chest movements change the distances between the ECG electrodes and therefore affect the morphology of QRS-complexes. Based on these changes, respiration can be measured indirectly from the ECG signals: ECG-Derived Respiration (EDR).

M. Schmidt et al. [18] summarized several EDR methods. One widely used method is based on R-wave amplitude (R peaks) change analysis: R-peak amplitude follows the respiration cycles and can be used to detect respiration phases, including apnea.

To further enhance the classification results while using only TS, we hypothesized that combining the time and frequency domain detections would provide more accurate results. We also made the assumption that cardiac sounds embed the same kind of information as classical EDR. Indeed, the phonocardiogram (PCG) is caused by the closure of the heart valves. There are two fundamental heart sounds, called S1 and S2. The period from S1 to S2 is systole, while the period from S2 to S1 is diastole. We call this signal, which has only been used for heart rate estimation [10,19], PCG-derived respiration (PDR). We show that detection can be improved by combining the detection results from the temporal and frequency detection domains and PDR. The remainder of this paper, thus, presents the combined algorithm and the new method to extract respiration information from cardiac sounds. We validated the algorithm through a trial and discuss the results.

## 2. Material and Method

### 2.1. Material

**Figure 1:**
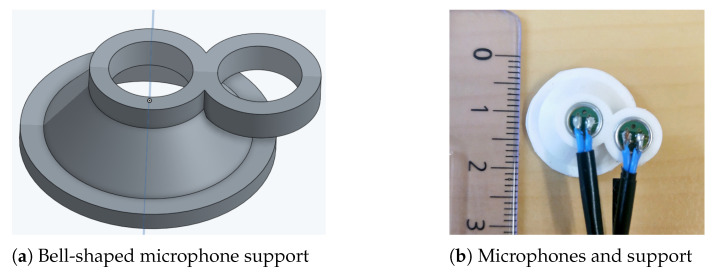
Illustration of the microphone setup.

**Figure 2:**
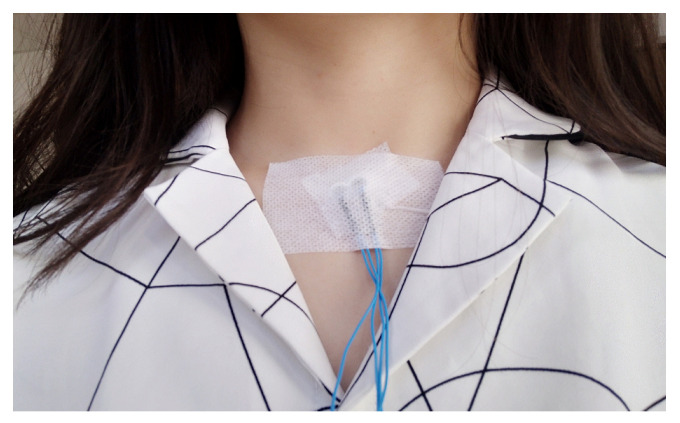
Position of the microphones: two microphones are inserted into the support that is fixed at suprasternal notch with two strips of adhesive tape.

Two uni-directional microphones (Pro-signal, ABM-708-RC) were used to record sounds: one for TS and one for environmental noise. The recorded environmental noise was intended to be applied to further reduce noise in the recorded TS, but it was not used in the present study (details in discussion). The microphones were inserted into a bell-shaped support (Figure 1a, design inspired by Reference [8]). The two microphones were located next to each other as illustrated in Figure 1b. During manipulation, only the support was fixed on the neck at the suprasternal notch with two strips of adhesive tape (as shown in Figure 2).

Each microphone was connected to an inhouse-designed analog card, which filters and amplifies recorded sounds from 100 to 1200 Hz with a second-order Band-Pass Butterworth filter and an amplification of 230 times. The filtering narrows the useful band to that known to contain the respiratory and cardiac sounds [8]. Last, the pre-processed sounds were sampled at 10 kHz with a PowerLab 16/35 (ADInstruments, Dunedin, New Zealand).

In order to evaluate the detection results of the proposed method, a low-resistance pneumotachograph (Hans Rudolf, 3700 series, linearity range 0–160 L/min; Kansas City, MO, USA), an abdominal strap (plethysmograph, AD Instrument/Pneumotrace II, UFI, Morro Bay, CA, USA), and three Ag/AgCl ECG electrodes (amplified with a Bio Amp: FE132, AD Instruments, Castle Hill, Australia) were also synchronously acquired with PowerLab. The algorithm was developed with MATLAB (Natick, MA, USA).

### 2.2. Protocol

Thirteen healthy participants were enrolled (5 women and 8 men; age: 36±10.37 years, weight: 66±12.27 kg, height: 172±7.84 cm). The study was conducted according to the principles of the Declaration of Helsinki and approved by the French Comité de Protection des Personnes Sud-Ouest & Outremer II, decision #2-18-12-MS1, 5 October 2018. The participants received detailed information about the study objective and methods and provided written informed consent.

**Figure 3:**
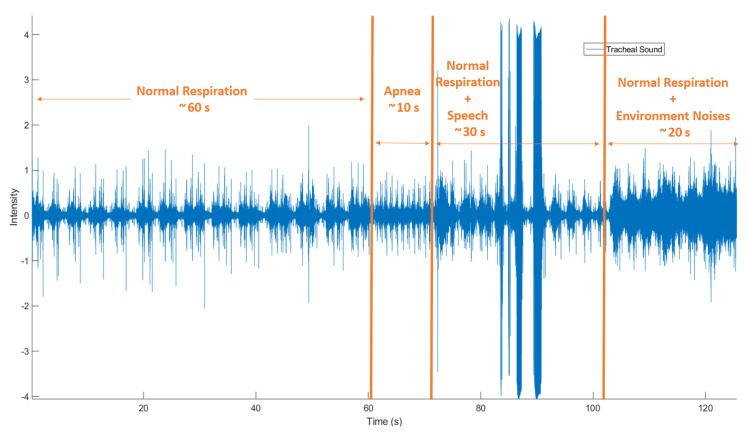
Recording procedure: 0–60 s normal respiration (NR); 60–70 s apnea (holding breathing spontaneously, AP); 70–100 s normal respiration + speech (RS); 100–120 s normal respiration + environmental noise (RN).

Each underwent two recordings of 2 min each: one in sitting position and the other in lying position. The 2-min sessions consisted of four parts: (1) normal respiration for 60 s, (2) apnea (spontaneous breath holding) for the next 10 s, (3) normal respiration while speaking for the next 20 s, and (4) normal respiration with strong environmental noise (a video with city traffic noise played right next to the microphones [20]) to the end. An example of session recording is shown in Figure 3, and all the examples shown in the next sections were extracted from this session.

### 2.3. Algorithm

In our previous studies, we developed and showed the feasibility of one first respiration detection algorithm tested on healthy subjects [21] and on one patient under implanted phrenic nerve stimulation [22]. This first algorithm was based on patient-specific thresholds and tested with short recordings. On the contrary, the present work used non-patient-specific thresholds and also included the new PDR.

**Figure 4:**
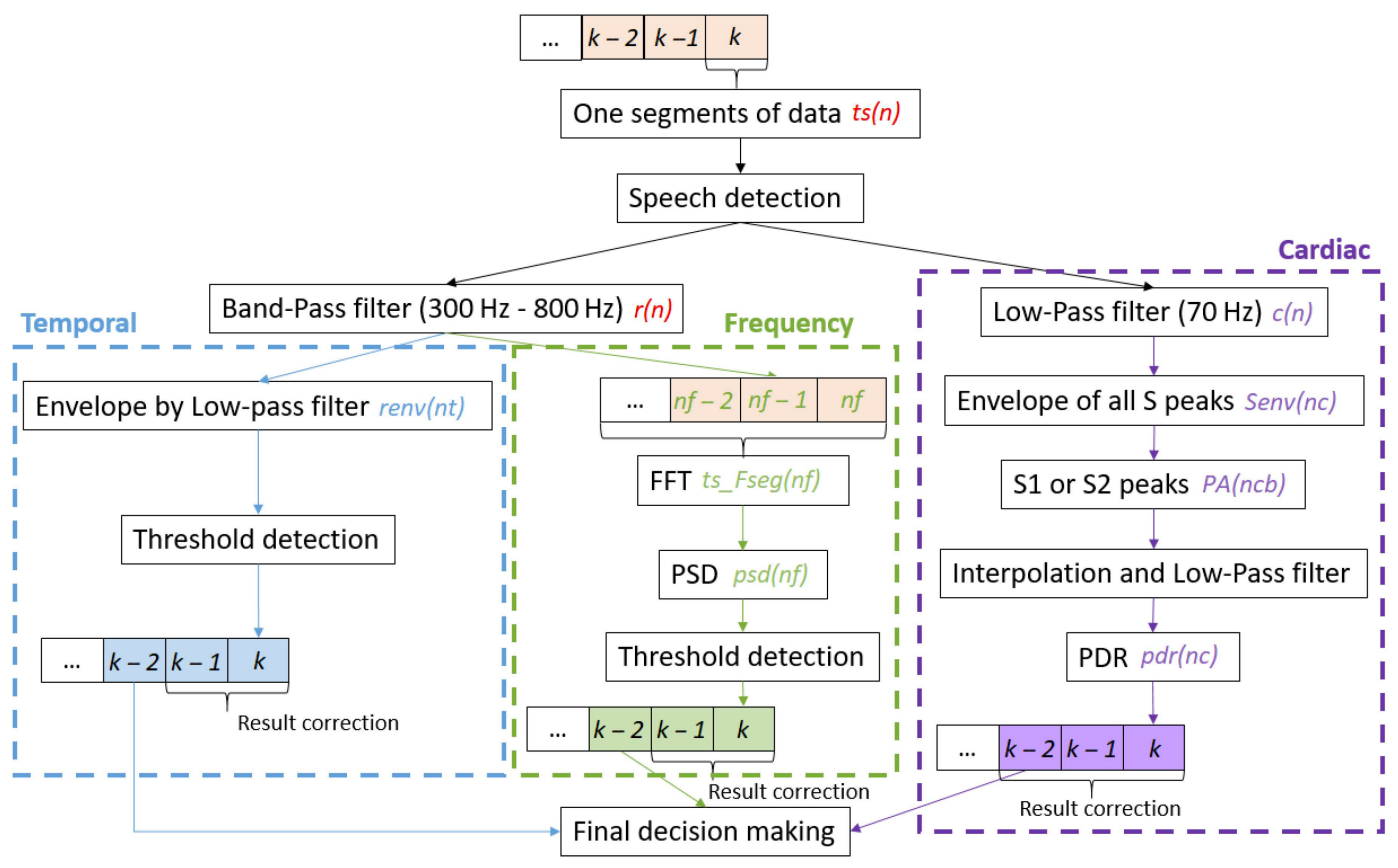
Diagram of the detection algorithm.

Recorded tracheal sounds (ts(n)) were processed in the temporal and frequency domains and also by the PDR method. The recordings were split into bins of l_seg = 16,384 sample (1.6384 s) duration. This is convenient for the Fast Fourier Transform (FFT) algorithm and enables the recording of at least one cardiac sound within each interval, assuming a heart-rate of minimum 38 beats per second (bps). The global algorithm is shown in Figure 4.

#### 2.3.1. Speech Detection

Speech or snoring can be considered as the expirations with the strongest intensity. These sounds have a much higher intensity, and a simple detection based on a threshold on the Mean Absolute Value (MAV) was performed:

$$MAV\left(k\right)=\frac{\sum_{n=k\times l\_seg+1}^{(k+1)\times l\_seg}\left|s(n)\right|}{l\_seg}\;,$$

where *k* is the index of the current bin. If this MAV(k) exceeds 0.5, it indicates that speech/snoring has occurred during this bin.

#### 2.3.2. Frequency Band Separation

**Figure 5:**
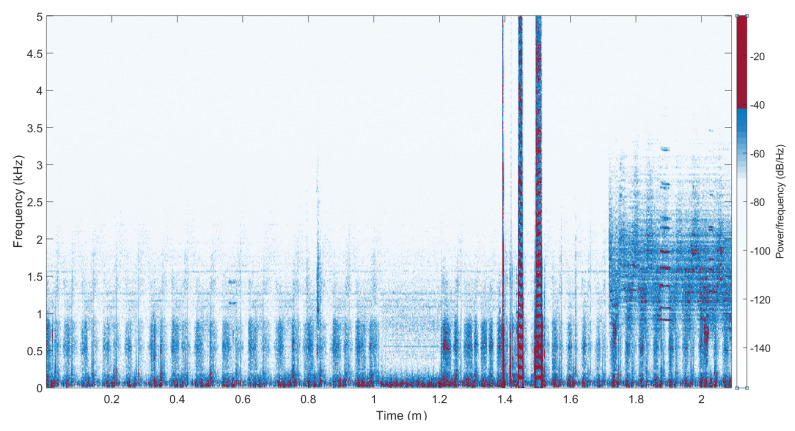
The spectrogram for one recording.

Respiratory sounds are usually centered between 20 Hz and 2000 Hz, but this may differ depending on the acquisition system [23]. For our recording system, the respiratory frequency content was mostly centered between 100 Hz and 1500 Hz (Figure 5). Furthermore, assuming environmental noise at low and high frequencies just above 1200 Hz, we designed a steep band pass filter with an eighth-order Butterworth from 300 Hz to 800 Hz to extract the respiratory signal (r(n)). To extract the cardiac sound signal, we designed a steep low-pass filter that extracted its most significant part, an eighth-order Butterworth low-pass filter at 70 Hz to get the phonocardiogram (c(n)).

#### 2.3.3. Temporal Envelope Detection

The temporal envelope (renv(n)) of the respiratory signal was obtained by applying a second-order low-pass Butterworth filter at 0.8 Hz on the rectified signal $\left|r(n)\right|$. Then, two thresholds were applied on renv(n):

1. The minimum threshold:
  th_T_min=110%×renv(3_s_apnea),
  where the renv(3_s_apnea) is renv(n) within any 3 s of apnea in the same recording;
2. The adaptive threshold:
  $$th\_T\_adapt\left(k\right)=90\%\times \frac{\sum_{n=k\times l\_seg+1}^{(k+1)\times l\_seg}renv\left(n\right)}{l\_seg}.$$

The result of the temporal envelope detection (detect_T(n)) was fixed to three states, presented in Table 1.

**Figure 6:**
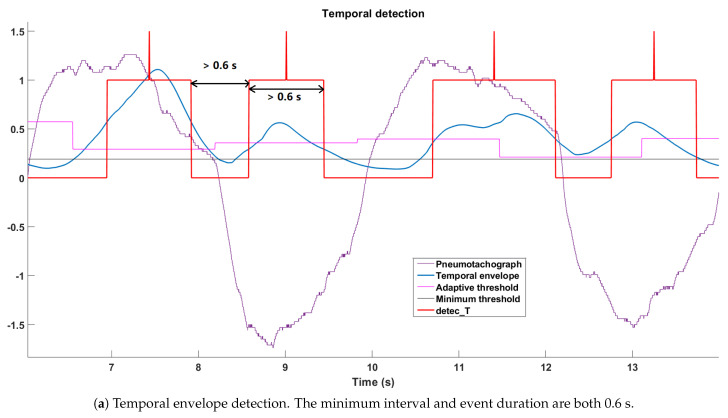

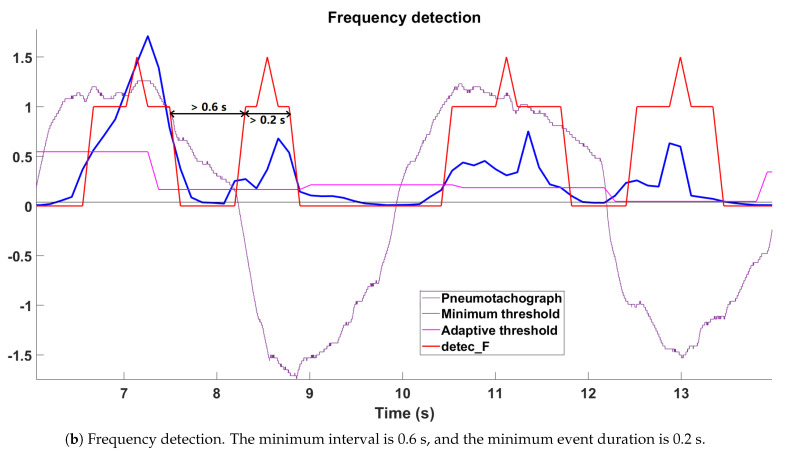
Temporal and frequency detection result of the same period from the same recording. A positive (resp. negative) pneumotachograph reference signal (purple) indicates inspiration (resp. expiration). The adaptive (pink) and minimum thresholds (gray) are marked in the figure. The envelope signal (temporal or PSD) is in blue, and the final detection result is in red with the peak “middle-timing moment”.

If detec_T=1 was obtained for bin *k*, an error correction method was applied on bins k−1 and *k*: a detected respiratory event was eliminated if it lasted less than 0.6 s because its duration was not in the normal range for an inspiration/expiration; detec_T(n) was forced to “0” if the interval from the previous detected event was less than 0.6 s. The middle-timing moment of a detected event was labeled (detec_T(n)=1.5) to indicate the position of this event to serve for the final decision-making. A temporal detection example is shown in Figure 6a, where the pneumotachograph signal (in purple) is considered the reference, a positive signal indicates an inspiration, and a negative one indicates an expiration. We also plotted the temporal envelope (in blue), the adaptive threshold (in pink), and the minimum threshold (in gray). The final temporal detection result is in red with the peak “middle-timing moment”. The minimum interval and event duration are both 0.6 s.

#### 2.3.4. Frequency Detection

In the frequency domain, one bin *k* of tracheal sounds ts(n) was divided into segments of 2048 samples (15 segments per bin). An FFT was processed on these segments with a Hanning window and 50% overlap (TS_Fseg(nf)). According to our recordings and frequency analysis, the useful frequency content was indeed between 400 Hz and 700 Hz: the PSD of this band was calculated (psd(nf)) as the frequency envelope. The detection part was similar to that described for the temporal envelope detection. Two thresholds were applied on psd(nf):

1. The minimum threshold:
  th_F_min=110%×psd(3_s_apnea),
  where the psd(3_s_apnea) is psd(nf) within any 3 s of apnea;
2. The adaptive threshold:
  $$th\_F\_adapt\left(k\right)=90\%\times \frac{\sum_{nf=k\times nb\_seg+1}^{(k+1)\times nb\_seg}psd\left(nf\right)}{nb\_seg},$$
  where nb_seg is the number of segments per bin, which equals 15.

Like detec_T(n), the frequency detection signal detec_F(nf) also had three states, as presented in Table 2, and the middle-timing moment of detected events was labeled as detec_F(nf)=1.5. For the error correction, the interval of each respiratory event should be longer than 0.6 s, and the minimum duration of each respiratory event is 0.2 s (illustrated in Figure 6b).

#### 2.3.5. PDR Detection

For the extracted cardiac signal, the MAV of the cardiac sound was computed every 0.06-s window (600 samples) with 0.03 s (50%) overlapping to obtain an envelope of S1 and S2 (Senv(nc)) [24].

**Figure 7:**
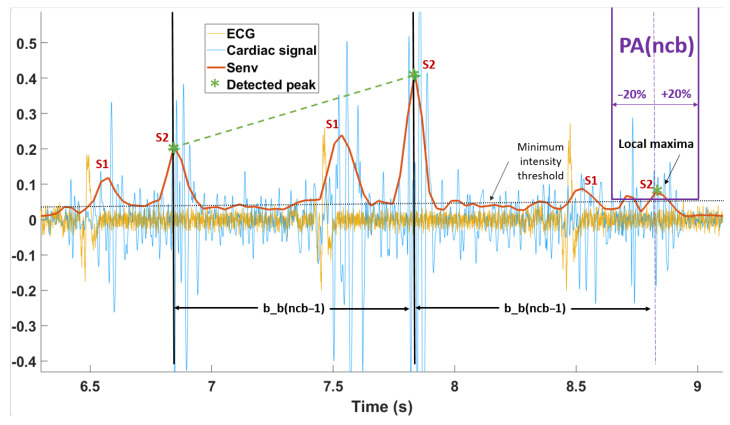
Cardiac S peak detection. There is a small delay between the electrocardiogram (ECG) (**yellow**) and the extracted cardiac signal (**blue**). Predicting area PA(ncb) (purple) defined by ±20% of last heart beat-to-beat interval b_b(ncb−1) and the adaptive threshold. The cardiac envelogram is in red, and the detected cardiac peak (S1/S2) is marked by a green star.

Inspired by the method of Kalkbrenner et al. [10], the detection of S1 or S2 was more robust in that the time intervals between these events could be bounded, as illustrated in Figure 7. Time boundaries were defined as follows: where pa_tmin and pa_tmax are borders of this area in the time axis, based on the previous beat-to-beat interval marked with a black line (in Figure 7) and defined with the following conditions:

$$\begin{array}{cc} \; & pa\_tmin(ncb)=80\%\times b\_b(ncb-1),\\ \; & pa\_tmax(ncb)=120\%\times b\_b(ncb-1). \end{array}$$

b_b(ncb−1) is the previous beat-to-beat time interval. Moreover, the lower boundary is limited to 0.4 s (150 beats per minute (bpm)) and the higher to 1.2 s (50 bpm). The local maxima in the predicted area PA(ncb) were considered the cardiac peak if its amplitude was higher than the adaptive threshold:

$$\begin{array}{cc} \; & pa\_adap=2\times mean(Senv(nc)). \end{array}$$

At the same time, the pa_adap should be greater than the minimum intensity threshold presented with a black dotted line in Figure 7 and defined as:

$$\begin{array}{cc} \; & pa\_min=30\%\times mean(Senv(first\_10\_seconds)), \end{array}$$

where Senv(first_10_seconds) is the cardiac envelogram for the first 10 s of recording. If there was no matching peak in this area, PA(ncb) was moved forward two samples to verify another local maxima.

A first-order interpolation between each detected peak (green dotted line in Figure 7) was applied, and the signal was upsampled at the same frequency of Senv(nc). Then the signal was filtered at 0.25 Hz by a second-order low-pass Butterworth filter. The filtered signal represents the upper envelope of S1 (or S2). Our hypothesis was that its variation would be due to respiratory effort (changes in thoracic volume): an increase in the signal would indicate an inspiration, while a decrease indicates an expiration. To reflect the correlation between the reference signals and the variation in the envelogram, we further assumed that its derivative was representative of the respiration phases as it was linked to the variation in the S1/S2 peak envelogram. We called this new curve PDR: phonocardiogram-derived respiration (pdr(nc)), indicating an inspiration when it is positive and an expiration when it is negative.

**Figure 8:**
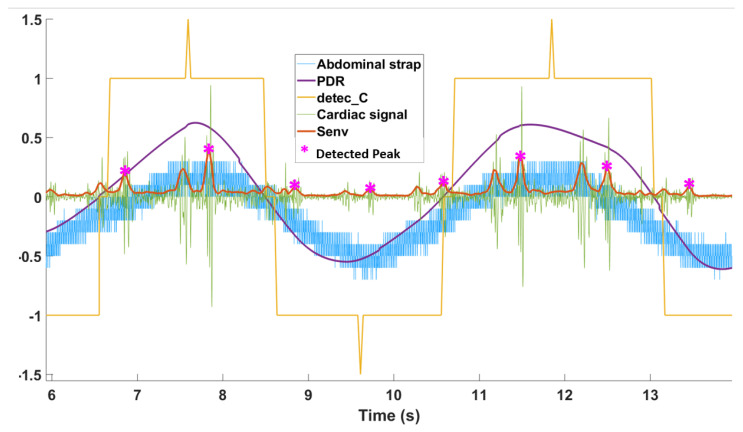
PDR. The extracted cardiac signal is shown in green, and the processed envelogram is in red. Only one cardiac peak (S1/S2) detected (**pink star**) for each cardiac cycle. Positive (Negative) of the PDR detection result (**yellow**) indicates the inspiration (expiration) as reference signal.

Unlike the two previous detection domains, the PDR signal (pdr(nc)) was able to distinguish inspiration and expiration. Thus, the cardiac (PDR) detection signal detec_C(nc) had four states as presented in Table 3, and the middle-timing moment of detected events were labeled as detec_C(nc)=1.5. If there were continuous inspirations/expirations, they were merged because usually inspiration and expiration alternate. The respiratory effort signal showed few respiratory pauses (about 0.2 s) or long duration respiratory events, although some detected event durations could last more than 3 s. Thus, the error correction was applied on three bins: *k*, *k* − 1, and *k* − 2. This made a processing delay of about 3.2 s (2 bins), which is acceptable according to the definition of apnea (10 s of absence of air flow). An example of cardiac detection is shown in Figure 8. The minimum respiratory event interval becomes 0.1 s, and minimum respiratory event duration fixes as 0.7 s.

#### 2.3.6. Decision-Making

The temporal and frequency envelope detected the presence of airflow (apnea or not), whereas PDR also assesses respiratory efforts to distinguish between central and obstructive apnea. The final detection was the fusion of the three detection domains. To detect apnea alone or the complete set of respiration phases, two decision-making methods are defined:

##### Apnea Detection

The parameter *Q* is used to evaluate the respiratory status and was calculated by the weighted average as follows:

$$\begin{array}{cc} Q=\; & \;1.5\times \frac{\sum_{nt=1}^{NT}detec\_T\left(nt\right)}{NT}+1.5\times \frac{\sum_{nf=1}^{NF}detec\_F\left(nf\right)}{NF}\\ \; & \;+0.5\times \frac{\sum_{nc=1}^{NC}\left|detec\_C(nc)\right|}{NC} \end{array}.$$

For the bin k−2, the final decision (detec(k−2)) is made as:

$$detec\left(k-2\right)=\left\{\begin{array}{c} 1\to respiration\;if\;Q\geq 1.5\\ 0\to pauses\;if\;Q<1.5\\ 2\;if\;speech/snoring\\ -1\to apnea\;if\;pauses>10s \end{array}.\right.$$

**Figure 9:**
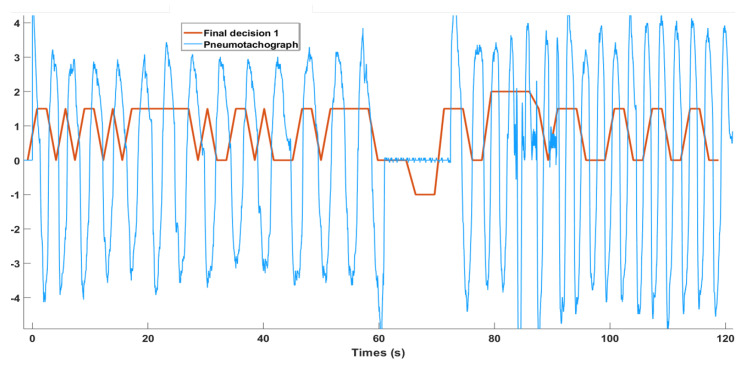
Final decision-making for apnea detection. Detection stat: 2—Speech; 1.5—Respiration; 0—Pauses; −1—apnea. The reference signal: pneumotachograph is in blue, and the apnea detection signal is in red.

An example is shown in Figure 9. In theory, the PDR is used to evaluate respiratory effort. However, when the signal-to-noise ratio is low because of weak respiratory sounds, PDR detection may have a better result than those obtained in the temporal and frequency domains. Nevertheless, most of the time, temporal and frequency domain detections were more sensitive, so the PDR detection results are less weighted.

##### Breathing Activities Assessment

**Figure 10:**
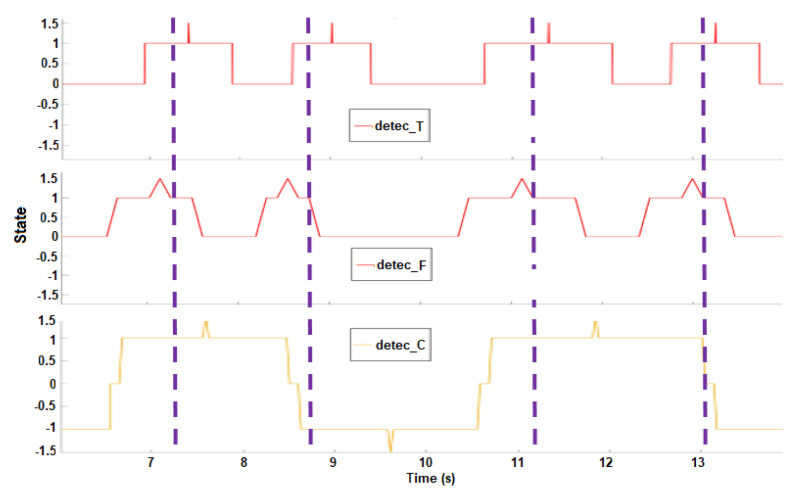
Final decision-making for breathing activities assessment. (**Top**): results from temporal detection. (**Middle**): results from frequency detection. (**Bottom**): results from cardiac (PDR) detection. The final detected events are marked in purple dashed lines.

The second algorithm detects the presence of each respiratory event. A respiratory event was considered to be detected if it was detected in at least two of the three detection domains, and the intervals between them were less than 1 s estimated with their middle-timing peak distances. An example is shown in Figure 10.

##### Statistics

Several means comparing statistical tests were applied to show the influence of speech, noise, and participant position. The compared data were: (1) specificity, sensitivity, and accuracy (SSA) during normal respiration+apnea (NR+AP) versus normal respiration + speech + normal respiration + environmental noise (RS+RN) (Figure 3), (2) SSA from sitting versus lying, (3) SSA of each detection domain versus the final combined results, (4) correlation between PDR and EDR, and (5) correlation between S-S and R-R intervals; all compared data were paired. Based on the Shapiro-Wilk test, if the data were normally distributed, the paired Student test was applied to compare means; otherwise, the Wilcoxon test was applied to compare medians. If the *p*-value was ≥0.05, we concluded that the difference between the two paired samples was not significantly different.

## 3. Results

### 3.1. Evaluation Method

**Figure 11:**
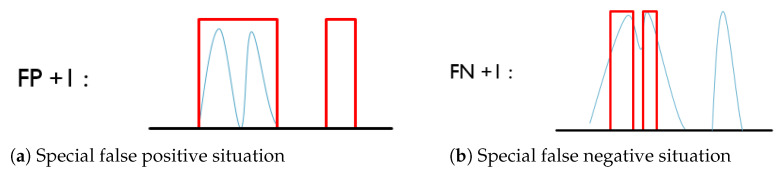
Example of breathing activity counting. Respiratory envelope (in blue), detection signal (in red). (**a**) If two continuous breathing activities are recognized as a single event, or the pause/apnea is detected as one breathing activity, a false positive detection will be reported; (**b**) if one breathing activity is recognized as two events, or one breathing activity is not detected, a false negative detection will be reported.

The proposed algorithm was applied on a total of 26 recordings of 2 min from 13 healthy participants, 13 in sitting and 13 in lying positions. All speech episodes were detected successfully in all the recordings. According to the two final decision-making methods, the respiratory detection results were evaluated on: (1) the ratio of apnea detection, and (2) the performance of breathing activities assessment with specificity, sensitivity, and accuracy, which are defined as follows:

$$\begin{array}{cc} \; & Specificity=\frac{TN}{TN+FP},\\ \; & Sensitivity=\frac{TP}{TP+FN},\\ \; & Accuracy=\frac{TP+TN}{TP+TN+FP+FN}, \end{array}$$

where TN, FP, TP, and FN are, respectively, the number of true negatives, false positives (Figure 11a), true positives, and false negatives (Figure 11b).

In our context, the detection system must not detect breaths that do not occur during apnea, i.e., FP. Given the definition of apnea, which is an absence of respiratory flow for at least 10 s, and the observation that the respiratory rate is on average 15 cycles per minute, this leads to:

- the specificity of respiratory detection (breathing/apnea) from TS must be >82.9%.
- the accuracy of respiratory detection (breathing/apnea) from TS must be >90.5%.

The FN are less important for patient safety. Given that the sensitivity is inversely proportional to FN, we fixed a minimum of 80% corresponding to <25% of FN compared to TP.

### 3.2. Apnea Detection

The rate of successful apnea detection was 92.3%. Among the 26 recordings that included a single apnea phase, two were missed and detected as long pauses because of unexpected environmental noise. In seven recordings, one or two more apneas were reported because the recorded respiratory sounds were too weak.

### 3.3. Breathing Activities Assessment

This method detects all breathing phases, including apnea. If only apnea detection is looked at, the success rate was 88.5%, slightly less than the previous method (92.3%).

**Figure 12:**
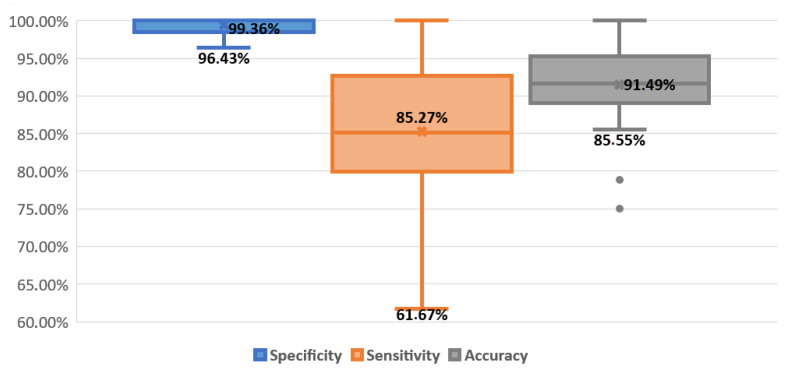
Box plot for final respiratory detection result. The line and the cross inside the box correspond to the median and the mean value, respectively. The box-limits represent the first and the third quartiles, the points highlight outliers, and the upper and lower bars represent the maximum and minimum values, respectively. Specificity: blue box. Sensitivity: orange box. Accuracy: gray box.

Looking at all breathing activities, for all 26 recordings, the average of these detection results (Figure 12) is 99.4% for specificity (min 82.9%), 85.3% for sensitivity (min min 80%), and 91.5% for accuracy (min 90.5%), which meets the minimum performance required for the system (described in Section 3.1). In more detail, all 26 recordings meet the required specificity, 19 out of 26 meet the required sensitivity, and 18 out of 26 meet the required accuracy.

#### 3.3.1. Influence of Speech and Noise

**Figure 13:**
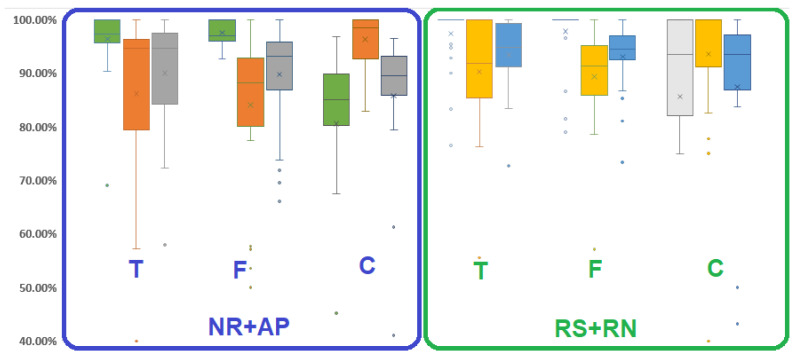
Influence of speech and noise on respiratory detection. Box plot (as box plot of Figure 12) for specificity (green and light gray boxes), sensitivity (orange and yellow boxes) and accuracy (dark gray and blue boxes) of temporal (T), frequency (F), and cardiac (C) detection domains, separated in NR+AP phases (in blue square) and RS + RN phases (in green square).

The influence of speech and noise on the detection results is presented in Figure 13. Comparing the results of the NR + AP versus RS + RN phases, only the specificity in the frequency domain and cardiac detection domain were better during the RS + RN phase (p<0.05, Table 4); no other parameters showed significant differences. This shows that noisy environments did not alter the detection results.

#### 3.3.2. Influence of Position

**Figure 14:**
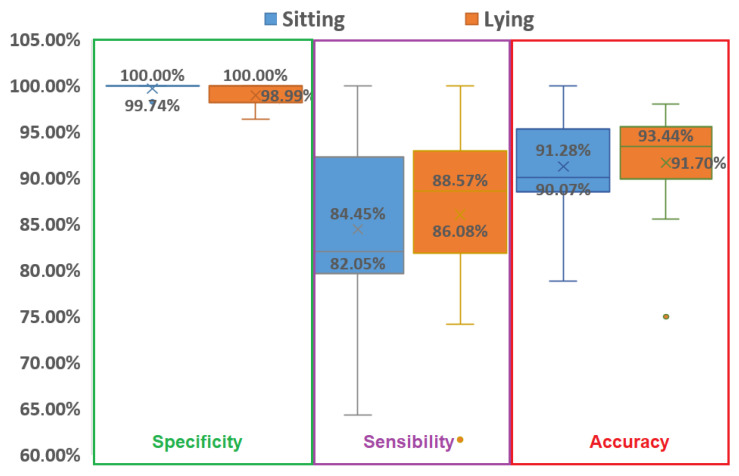
Box plot (like box plot of Figure 12) for specificity (green rectangle), sensitivity (purple rectangle), and accuracy (red rectangle) in sitting (orange boxes) and lying (blue boxes) positions.

Further examination of the detection results in the different participants positions were compared (Figure 14) The specificity, sensitivity, and accuracy showed no significant differences between sitting and lying positions (Table 5).

#### 3.3.3. Advantage of Combining Several Detection Domains

**Figure 15:**
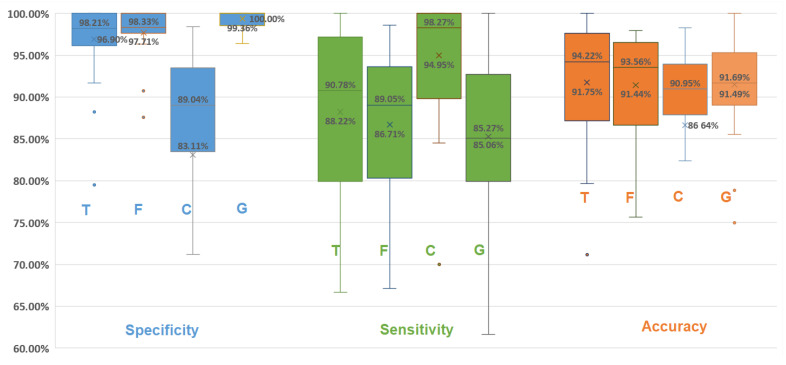
Box plot (as box plot of Figure 12) for specificity (blue), sensitivity (green), and accuracy (orange) of temporal (T), frequency (F), and cardiac (C) detection domains, as well as global result (G).

The SSA of each detection domain and the global results (combined results from the 3 domains) are shown in Figure 15. Specificity was significantly improved (reduced false positives) with the combined detection approach compared to single domain detection (Table 6). Sensitivity was significantly reduced (increased false negatives) with the combined detection approach compared to PDR detection. In all other comparisons, there was no significant difference.

### 3.4. Cardiac Sound Processing

As the PDR method is a new approach, we validated S1/S2 detection against classical R-peak detection from ECG recordings: R-peaks were detected through threshold detection and manually verified. In general, there should be one S1/S2 complex for each cardiac cycle corresponding to one R-peak. Both the reliability of the S-S interval (versus R-R interval) and PDR versus EDR (envelope of R-peak amplitude) were assessed.

#### 3.4.1. Correlation between PDR and EDR

The obtained PDR was compared with EDR because they both resulted from thoracic volume changes due to respiration.

**Figure 16:**
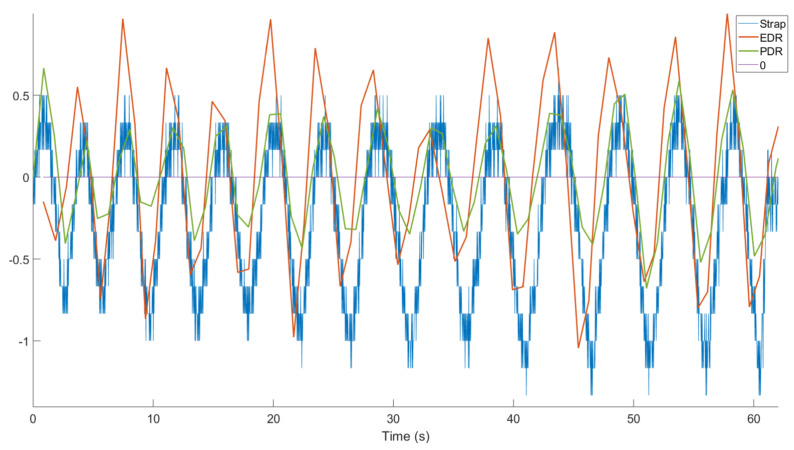
PDR (green) processed from tracheal sounds and ECG-Derived Respiration (EDR) (red) processed from ECG and the thoracic strap signal (blue). Their positive (negative) slopes indicate inspiration (expiration).

**Figure 17:**
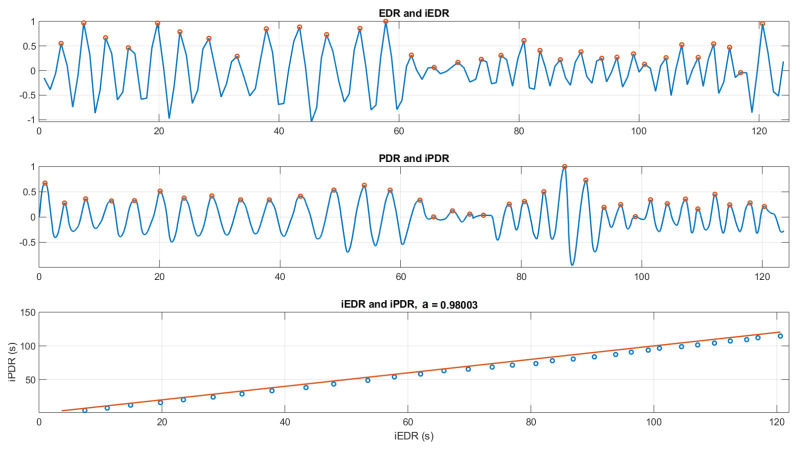
(**Top**): the EDR signal and its local maxima (peaks) iEDR. (**Middle**): the PDR signal and its local maxima iPDR. (**Bottom**): the linear regression fit line between iEDR and iPDR (a=0.98).

For the example shown in Figure 16, the amplitude variations of EDR versus PDR were obviously not correlated and thus PDR cannot be used for assessing the quantitative volume of respiration. However, both signals seemed to oscillate in phase: we thus compared the respiration cycles in the PDR vs. EDR signals. To do so, local maxima of each curve (iPDR(k),iEDR(k)) were detected and their timing compared (Figure 17).

A perfect match between the two should lead to a linear equation, such as

**Figure 18:**
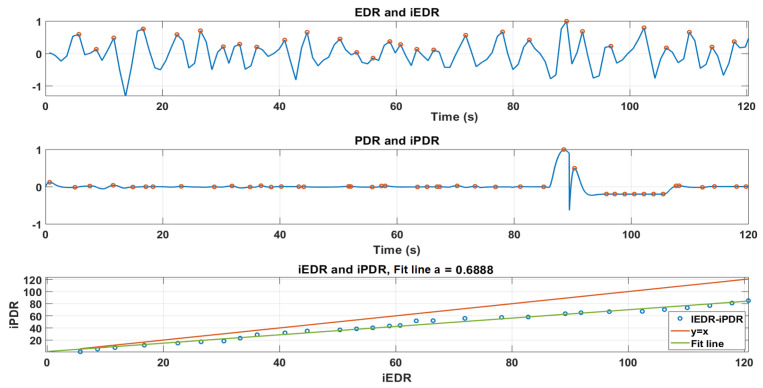
(**Top**): the EDR signal and its local maxima (peaks) iEDR. (**Middle**): the PDR signal and its local maxima iPDR. (**Bottom**): the theory fit line(y = x) in red and the linear regression fit line between iEDR and iPDR (a=0.68).

iPDR(k)=a×iEDR(k),

where *a* in theory is equal to 1. Indeed, when iPDR(k) and iEDR(k) are not matched (missed, shifted, or additional peaks), it results in *a* variation (a≠1, (Figure 18). Thus, the slope of the first-order linear regression fit line (*a*) could be a good indication to evaluate the correlation between EDR and PDR. Essentially, the closer slope (*a*) is to 1, the more PDR and EDR are correlated.

**Figure 19:**
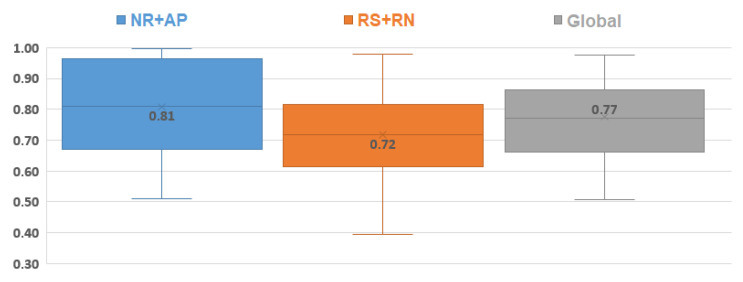
Box plot (like box plot of Figure 12) for the evaluation of the results of PDR according to EDR, separated into NR+AP (blue), RS+SN (orange), and global results (gray).

The result of all recordings is illustrated in Figure 19, separated into the NR + AP phase, RS + RN phase (as described in Figure 3), and global results. The global mean slope was 0.77, which shows a good correlation between EDR and PDR. The Wilcoxon test (*p* = 0.0067) showed that the results during NR + AP were better than the ones during RS + RN because the cardiac peaks were difficult to detect during speech.

**Figure 20:**
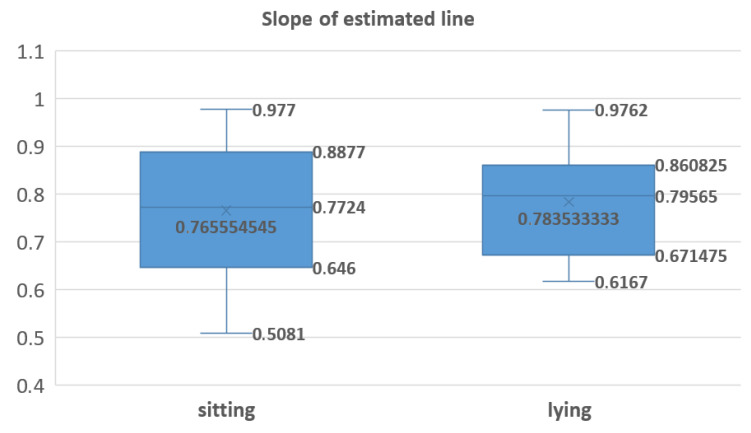
Box plot (like box plot of Figure 12) for the evaluation of the results of PDR according to EDR, separated in sitting (**left**) and lying (**right**) positions.

The PDR results were also evaluated in sitting and lying positions (Figure 20), and the Student test showed no significant difference in the means (*p* = 0.5359).

#### 3.4.2. Heart Beat-to-Beat Interval

The heart beat-to-beat intervals derived from cardiac sounds were compared to the heart rate variation (HRV) (R-R interval) calculated from ECG. For each recording, the result was also separated into NR + AP and RS + RN. The ideal relationship should be a straight line y = offset: the initial offset is due only to the delay between R-peak and S sounds. However, the small oscillations are explained by the detection of either S1 or S2. The NR + AP phase showed a good fit, but the introduction of noise led to strong S complex mislocations.

Thus, plotting S-S versus R-R intervals showed mainly four groups of points corresponding to R-R vs. S1-S1 (or S2-S2), R-R vs. S1-S2, R-R vs. S2-S1, and outliers when the S1-S2 complex was missed. In the latter case, all the S-S intervals greater than two times the R-R interval were considered outliers and thus removed: a ratio $R_{error}$ was calculated as:

$$R_{error}=1-\frac{N_{abnormal}}{N_{total}},$$

where $N_{abnormal}$ is the number of outliers, and $N_{total}$ is the total number of points. This ratio was used to weight the final assessment. Once outliers were removed, a first fit line slope (β) was estimated using the solution of: $\underline{SS}=\beta \underline{RR}$. Based on this line, two other shifted lines defined the group separation in considering the averaged S1-S2 interval (0.29 s) and minimum S2-S1 interval (0.5 s). Thus, two boundary lines and one central line are obtained:

$$\begin{array}{cc} \; & y1=\beta x+0.5,\\ \; & y2=\beta x,\\ \; & y3=\beta x-0.29. \end{array}$$

**Figure 21:**
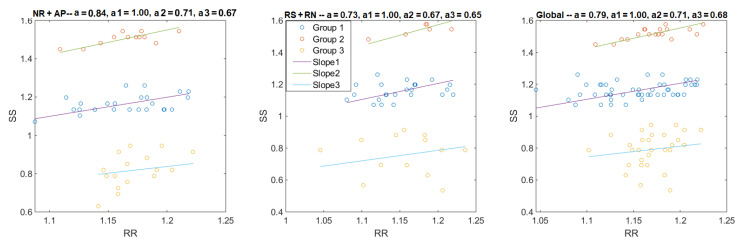
The left, middle, and right figures show the linear regressions for NR + AP, RS + RN, and the whole recording. The blue points are from the group of R-R vs. S1-S1 or S2-S2 (group 1, at the middle group of each figure), the orange points are from the group of R-R vs. S2-S1 (group 2, at the top of each figure), and the yellow points are from the group of R-R vs. S1-S2 (group 3, at the bottom of each figure). The slopes of each fit line are presented the in figure title (a1, a2, and a3 are slopes for the group 1, group 2, and group 3, respectively).

Thus, all points were then separated into three groups (by looking for their nearest line). As in the PDR and EDR assessment, the slope of the linear regression fit line was used to evaluate the S peak detection performance, three different fit lines could be calculated separately for these three groups of points, as in the example shown in Figure 21.

**Figure 22:**
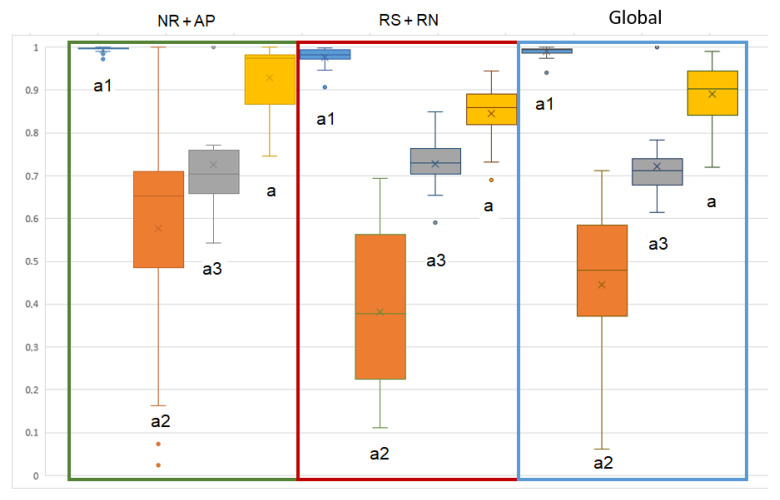
Box plot (like the box plot of Figure 12) for the distribution of fit line slopes for each group in each part and the global signal (in different rectangle).

The assessment was based on how close the slope is to 1 (the best theory slope) as follows:

$$a_{x}=1-\left|a_{x}^{\prime }-1\right|,$$

where $a_{x}^{\prime }$ is the slope of the fit line for group *x*, and $a_{x}$ is the new slope of group *x* to evaluate the correlation. Thus, an average slope was weighted as:

$$a=R_{error}*\frac{n_{1}*a_{1}+n_{2}*a_{2}+n_{3}*a_{3}}{n_{1}+n_{2}+n_{3}},$$

where $n_{x}$ presents the number of points in group *x*. For example, in Figure 21, the averaged slopes (*a*) for NR + AP, RS + RN, and the whole recording were 0.84, 0.73, and 0.79, respectively. The results for the 26 recordings are presented in Figure 22. The slope of group 1 in each part was very close to 1, which means a high correlation between the S-S and R-R interval lengths. The medians of the results from RS + RN and NR + AP were significantly different (*p* = 0.00004). The cardiac peaks were more difficult to detect from TS during speech or with environmental noise. Group 2 and group 3 showed even poorer correlations, but, despite their relatively small numbers, the global mean still reached 0.89. Thus, the S-S interval obtained from the proposed cardiac peak detection method is a reliable way to estimate heart rate or as an alternative method to present the heart rate variation (HRV).

## 4. Discussion

In this paper, we present an original method for continuous respiratory monitoring based on TS. The first aim was to detect apnea, and the second aim was to further classify breathing activities. Previous methods used a detection solution based on either the temporal features of sound or its frequency content. On the contrary, we combined classifications from the two domains with new signal processing based on cardiac sounds. We thus increased the performances of the classification. This preliminary method was proved to be feasible on 13 healthy subjects with 26 apnea events. For a better evaluation of the proposed algorithm, the next step is to validate on the main target population—patients with implanted phrenic nerve stimulation. Otherwise, we can underline the following outcomes.

Figure 15 and Table 6 show greater sensitivity with PDR than the final combined results. This is because the final decision-making method had, in fact, missed some events due to delays and different durations in the results of each detection domain (as shown in Figure 10), even though they were well detected in other domains. As a result, false negatives were added and the sensitivity was reduced. On the other hand, some false positives caused by noise could be eliminated in other detection domains (e.g., the temporal envelope could eliminate brief noises and the frequency domain might eliminate long duration noises with different frequency content than respiration). The number of false positives was reduced, and, therefore, the specificity was increased. Even though the sensitivity was ultimately reduced, it was still above the minimum requirement for a detection system (>80%), so the results remain acceptable. Furthermore, in this study, high specificity was more important than high sensitivity or high accuracy because false positives would mean that apnea had been detected as respiration. In other words, patients would stop breathing, but the detection system would not deliver an alarm.

This study used two microphones to record TS and environmental noise separately. The ideal would have been to use recorded noise to reduce the environmental noise in the recorded TS by using the Wiener adaptive filter [25]. But, in the obtained recordings, the noisy environment was simulated by playing specific videos, in which the frequency band was higher than the respiratory frequency band (Figure 5) so that it could be easily filtered. A better way would be to record data in a real noisy environment (e.g., on street) to see if the design with noise reduction by two microphones would improve the detection result.

To our knowledge, the proposed PDR method had never been studied before. In this study, however, we showed the feasibility of evaluating respiratory effort with PDR by comparing the result with EDR. This means that, using only a microphone, it is possible to continuously monitor cardiac activity, detect the apnea episodes, and eventually discriminate between the type of apnea based on the PDR (apnea with respiratory effort: obstructive apnea; otherwise: central apnea). In our study, PDR also allows to detect correctly respiration phases and apnea, in particular, when the respiration noise is very weak: in this case, time and frequency domains detection may fail, while PDR succeeds. It should, nevertheless, be noted that the EDR method is still not widely applied in clinic routine, and it is not known to be a good indicator of respiratory effort. More comparisons with a plethysmograph (PSG) are thus needed. In addition, the proposed algorithm has not yet been tested in individuals with real apnea. Thus, the next step will be to record patients’ TS synchronized with PSG to test whether the same algorithm still works correctly. Furthermore, cardiac peak detection may be improved by combining wavelet decomposition [26] (better detection of peak position) and the envelogram by absolute value [24] (better detection of peak amplitude).

Monitoring TS could be a useful way beyond the very limited niche of patients with diaphragm pacing or sleep apnea. Indeed, it would provide a non-invasive way to approximate inspiratory flow that would be useful in all patients requiring respiratory monitoring in acute situations (e.g., as a safety measure during the administration of morphine for acute pain) and in chronic situations (e.g., home mechanical ventilation).

## 5. Conclusions

The method we presented is able to continuously monitor healthy subject’s respiration (apnea detection, breathing activities assessment, respiratory effort evaluation) and cardiac activity (heart beat detection) using only a microphone recording tracheal sounds (TS). The next step will be to validate the approach on patients with implanted phrenic nerve stimulation. Recorded tracheal sounds were processed in temporal envelope detection, frequency envelope (PSD) detection, and PDR (cardiac) detection, and then combined for the results. The proposed algorithm was tested on 26 recordings from 13 healthy participants, in sitting and lying positions, and the apnea detection rate was 92.3%, with 99.36% specificity, 85.27% sensibility, and 91.49% accuracy, which meets the minimum requirements for the system. Indeed, noise, like speech or from the environment, may influence the detection result, but within an acceptable range. The different positions could lead to different recorded sound intensities but did not influence the detection result. Compared to using single detection domain, combining the three detection domains significantly improved the algorithm’s specificity, which is the most important parameter in apnea detection. But, it reduced its sensitivity (within an acceptable range) due to the decision-making method.

The new proposed method, phonocardiogram-derived respiration (PDR), showed a good correlation with EDR, which demonstrates the feasibility of using PDR to monitor respiration. At the same time, the comparison of R-R intervals and S-S intervals also indicated a good correlation; thus, it is also possible to monitor heart activity from PCG extracted from TS. More comparisons with PSG are, nevertheless, needed with patients for further confirmation of the method’s performance.

## Figures and Tables

**Table 1:** Temporal detection states.

State of detect_T(n)	Explication	Conditions
0	Pause/apnea	th_T_adapt(k)≤th_T_min
1	Inspiration/Expiration	renv(n)>th_T_adapt(k)
2	Speech/Snoring	MAV(k)>0.5

**Table 2:** Frequency detection stats.

State of detect_F(nf)	Explication	Conditions
0	Pause/apnea	th_F_adapt(k)≤th_F_min
1	Inspiration/Expiration	psd(nf)>th_F_adapt(k)
2	Speech/Snoring	MAV(k)>0.5

**Table 3:** Cardiac detection (phonocardiogram (PCG)-derived respiration (PDR)) stats.

State of detect_C(n)	Explication	Conditions
−1	expiration	pdr(nc)<0
1	Inspiration	pdr(nc)>0
0	Pause/apnea	eventduration<0.7 s orintervall<0.1 s
2	Speech/Snoring	MAV(k)>0.5

**Table 4:** Median values comparing for specificity, sensitivity and accuracy of each detection domain—NR + AP vs. RS + RN. * *p*-value < 0.05, difference between the two paired samples was significantly different.

Detection Domain	Median Comparison	*p*-Value
**Temporal**	Specificity	*p* = 0.0966
	sensitivity	*p* = 0.8829
	Accuracy	*p* = 0.3638
**Frequency**	Specificity	*p* = 0.0088 *
	sensitivity	*p* = 0.2032
	Accuracy	*p* = 0.1219
**Cardiac (PDR)**	Specificity	*p* = 0.016 *
	sensitivity	*p* = 0.7369
	Accuracy	*p* = 0.0851

**Table 5:** Median values comparing for specificity, sensitivity, and accuracy—sitting vs. lying.

Median Comparison	*p*-Value
**Specificity**	*p* = 0.2079
**sensitivity**	*p* = 0.7354
**Accuracy**	*p* = 1

**Table 6:** Median values comparing for specificity, sensitivity, and accuracy of each detection domain—single detection domain vs. combining detection domain. * *p*-value < 0.05, difference between the two paired samples was significantly different.

Median Comparison	Detection Domain	*p*-Value
**Specificity**	Temporal	*p* = 0.00889 *
	Frequency	*p* = 0.01489 *
	Cardiac	*p* = 9.896 × 10 ${}^{-6}$ *
**Sensitivity**	Temporal	*p* = 0.09933
	Frequency	*p* = 0.6348
	Cardiac	*p* = 0.001801 *
**Accuracy**	Temporal	*p* = 0.8417
	Frequency	*p* = 0.8028
	Cardiac	*p* = 0.2079

## Data Availability

Data sharing is not applicable to this article.
